# Multiple intraneural glomus tumors in scapularis region: A rare case report and review

**DOI:** 10.1097/MD.0000000000043032

**Published:** 2025-06-27

**Authors:** Huji Zhang, Hongyan Wu, Zhongjiao Chen, Bangfeng Zeng, Long Wang

**Affiliations:** a Department of Pathology, Guiqian International General Hospital, Guiyang, Guizhou Province, China; b Guizhou Medical University, Guiyang, Guizhou Province, China.

**Keywords:** multiple intraneural glomus tumors, scapular region, surgical resection

## Abstract

**Rationale:**

Glomus tumors (GTs) are painful and typically benign neoplasms found in the subungual region of the fingertips. However, the occurrence of multiple GT within nerve fascicles in the scapular region is rare.

**Patient concerns:**

A 41-year-old male presented with a 5-year history of progressive tenderness in the scapular region, accompanied by elevated local skin temperature. Magnetic resonance imaging revealed a small abnormal signal measuring approximately 3.1 × 3.0 mm, appearing hypoechoic on T1 and hyperechoic on T2 images, with clear boundaries. The lesion was initially diagnosed as an inflammatory lesion.

**Diagnoses:**

Multiple intraneural GT located within different nerve fascicles were diagnosed based on immunohistochemical analysis and hematoxylin–eosin staining.

**Interventions:**

The nodules were completely excised.

**Outcomes:**

One week later, the patient reported significant pain relief compared with preoperative symptoms.

**Lessons:**

To the best of our knowledge, this is the first documented case of multiple intraneural GTs occurring within 3 different nerve fascicles to highlight the importance of accurate diagnosis to prevent misdiagnosis.

## 1. Introduction

Glomus tumors (GTs) are typically located at the distal ends of the limbs and present as solitary lesions. These tumors exhibit a classic triad of symptoms: pain, tenderness to palpation, and cold hypersensitivity.^[1,2]^ With an incidence of <2% among soft tissue tumors, diagnosing GTs can be particularly challenging when they occur in uncommon locations.^[1–3]^ In this report, we present the first documented case of multiple intraneural GTs located in different nerve fascicles and aim to aid clinicians in avoiding misdiagnosis.

## 2. Case presentation

A 41-year-old Chinese man, without significant B symptoms, prior chronic medical conditions, and a family history of hereditary diseases, presented with severe pain near his left shoulder, which had persisted for the past 5 years. The pain was exacerbated by direct pressure and was associated with an elevated skin temperature in the same area. Upon physical examination, a palpable mass was detected in the left scapular region, exhibiting significant tenderness to touch, with the surrounding skin appearing red and swollen.

**Figure 1.**
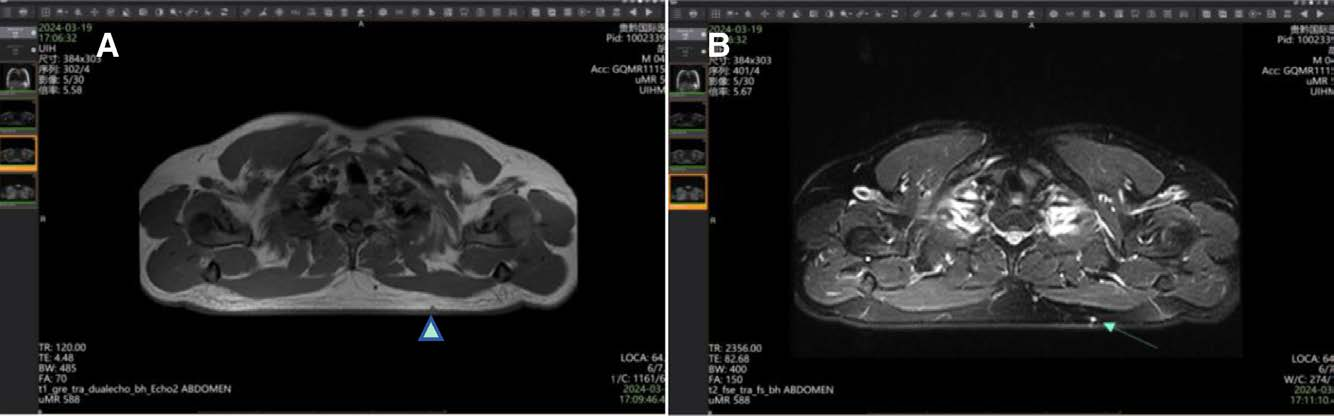
MRI revealed a small, abnormal signal measuring approximately 3.1 × 3.0 mm located beneath the subcutaneous fatty tissue in the left scapular region. The images displayed T1 hypointensity (A) and T2 hyperintensity (B), with a clear boundary observed around the lesion. MRI = magnetic resonance imaging.

Magnetic resonance imaging (MRI) revealed a small abnormal signal measuring approximately 3.1 × 3.0 mm, appearing hypoechoic on T1-weighted images and hyperechoic on T2-weighted images, with well-defined borders (Fig. 1). The lesion was initially interpreted as an inflammatory process. Laboratory studies showed normal results, and the patient denied any relevant medical or family history. Both the clinical presentation and MRI findings suggested the presence of inflammatory lesions.

**Figure 2.**
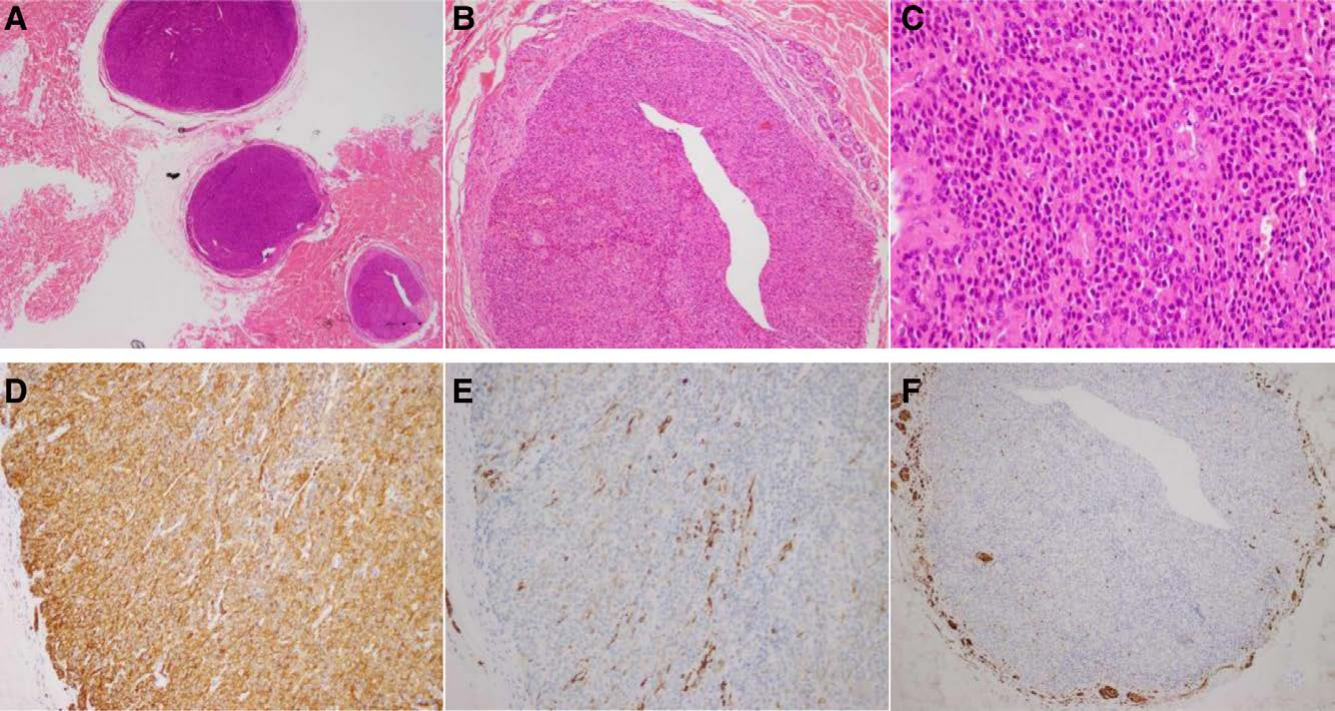
(A) On the low magnification (H&E, ×20), the mass was comprised of 3 well-circumscribed isolated nodules. (B) On the higher magnification (H&E, ×100), the tumor cells were encapsulated by the nerve fascicles. (C) Each nodule consisted of sheets, nests of tumor cells small in uniform, oval or spindle-shaped with round nuclei, and small vessels interspersed (H&E, ×400). (D) The tumor cells were positive for SMA diffusely with strong membranous accentuation. (E) Focally expression of desmin was in the tumors. (F) The nerve fascicles surrounding the tumor cells were highlighted positive for S100. H&E = hematoxylin–eosin, SMA = smooth muscle actin.

The nodule was surgically excised, located just beneath the subcutaneous fatty tissue, and was noted to be well-circumscribed. The excised tissue was sent for histopathological examination. Histological analysis revealed 3 well-defined isolated nodules encapsulated within different nerve fascicles, with diameters ranging from 1.5 to 4 mm. Each nodule consisted of sheets and nests of small, uniform tumor cells with centrally located round nuclei and amphophilic to lightly eosinophilic cytoplasm surrounding the vessels (Fig. 2A–C). Immunohistochemical (IHC) staining demonstrated that the tumor cells were diffusely positive for smooth muscle actin and focally positive for desmin (Fig. 2D and E), and had a low Ki67 labeling index of 1%. The tumor cells were negative for cytokeratin, Cga. S100 staining was negative within the tumor lesions but positive in the surrounding nerve fascicles (Fig. 2F). All IHC above were performed using Envision and Roche immunohistochemistry platforms.

The patient was diagnosed with multiple intraneural GTs. One week after the surgery, the patient reported a significant improvement in pain compared with his preoperative symptoms. Three months later, follow-up showed that the patient had a surgical scar on the back, and the pain symptoms had resolved.

## 3. Discussion

GTs are benign soft tissue neoplasms that can occur at any age and have a wide distribution. They are most commonly found in the fingertips and nail beds, but can also appear in rare instances such as the trunk, bone, and others.^[4,5]^ These tumors are characterized by classic symptoms, including paroxysmal pain, localized tenderness, and hypersensitivity to cold.^[3,4]^ However, extra-digital GT, such as those presented in this case, are less common and can complicate the diagnostic process due to their heterogeneous presentation.^[5–9]^ As a result, diagnosing GTs can be quite challenging, as they are often misdiagnosed as other more common tumors or inflammatory lesions.

To the best of our knowledge, only 3 cases of multiple intraneural GT have been reported to date.^[4,10,11]^ In these instances, the sex ratio was 3:1. In addition, 2 cases documented in the literature involved digital nerves, while 1 case was located on the right mid-back. We report the first case of extra-digital multiple intraneural GT occurring in 3 different nerve fascicles in the scapular region. The symptoms associated with multiple intraneural GT, akin to those of classic solitary GT, may include tingling, cold sensitivity, and paresthesia. Due to the small size of extra-digital nerve tumors and the variability of their symptoms, definitive diagnoses based solely on physical examination and clinical presentation are challenging, often leading to initial misdiagnosis as neuromas or inflammatory lesions.

Although ultrasound and computed tomography are commonly used, MRI is the most effective tool for pinpointing the location of lesions and the tissues from which they originate, particularly for detecting tumors as small as 2 mm in diameter, according to the literature.^[9,12,13]^ GT typically presents with low signal intensity on T1-weighted images and high signal intensity on T2-weighted images, resembling inflammatory lesions that lack clear boundaries. In the present case, although the tumor in the left scapular region exhibited classic MRI characteristics of GT with well-defined borders, clinicians and radiologists misdiagnosed it due to its rarity and the similarity of its MRI appearance to inflammatory lesions. While MRI may not always provide a definitive diagnosis, it remains a valuable tool for assisting surgeons in planning surgical excision.

Microscopically, multiple intraneural GT shares the same histopathological and IHC features as common GT, with the distinction of surrounding nerve fascicles. Glomus cells are small, uniform, oval, or spindle-shaped cells with centrally located round nuclei and amphophilic to lightly eosinophilic cytoplasm surrounding the vessels.^[1–4]^ IHC staining of GT typically shows positive smooth muscle actin expression with strong membranous accentuation, along with positive desmin and caldesmon. Although S100 is generally negative in tumor cells, the surrounding nerve fascicles of intraneural GTs exhibit S100 positivity. In the differential diagnosis, negative staining for cytokeratin and S100, insm, and so forth, in the tumor cells can rule out neuroendocrine tumors and neurofibromas, among other possibilities.^[1,4,14]^

While GTs and their variants are benign neoplasms, they can occasionally recur. Surgical excision remains the only feasible treatment, and the prognosis is excellent with early diagnosis and appropriate intervention. Some nerve fascicles associated with intraneural GTs may be too thin to be detected before pathological examination and can be inseparable from the tumors themselves, necessitating the removal of segments of the nerve fascicle along with the tumor. If functional nerves are involved, direct nerve repair should be performed to mitigate the impact of surgery on nerve function.^[15]^ In the present case, the nerve fascicles associated with the multiple intraneural GTs were terminal in the scapular region, rendering the consequences of nerve defects negligible. Intraneural GTs should be included in the differential diagnosis of local lesions, particularly in rare cases. This report emphasizes the importance of considering the possibility of multiple intraneural GTs at unexpected sites to avoid misdiagnosis and provide timely relief for patients.

## 4. Conclusion

In summary, this case report presents a rare but significant condition that underscores the need to avoid misdiagnosis due to its rarity and nonspecific clinical presentation. It serves as a reminder for clinicians to maintain a broad differential diagnosis and to utilize imaging and histopathological examinations to achieve an accurate diagnosis. Early surgical intervention can lead to favorable outcomes and significantly enhance the patient’s quality of life.

## Author contributions

**Writing – original draft:** Huji Zhang.

**Writing – review & editing:** Long Wang.

**Investigation:** Hongyan Wu.

**Resources:** Zhongjiao Chen, Bangfeng Zeng.
